# Conditions for communication between health care professionals and parents on a neonatal ward in the presence of language barriers

**DOI:** 10.1080/17482631.2019.1652060

**Published:** 2019-08-09

**Authors:** Katarina Patriksson, Stefan Nilsson, Helena Wigert

**Affiliations:** a Institute of Health and Care Sciences, Sahlgrenska Academy, University of Gothenburg, Gothenburg, Sweden; b Division of Paediatrics, NÄL Hospital, Trollhättan, Sweden; c Division of Neonatology, Sahlgrenska University Hospital, Gothenburg, Sweden

**Keywords:** Communication, families, field study, immigrant, language barriers, neonatal

## Abstract

**Purpose**: Family-centred neonatal care views parents and child as a unit, and aims to support each family on the basis of its specific needs. Good communication can increase parents’ satisfaction and reduce tension, and is necessary to create a mutual trustful relation, but is influenced by language barriers. We aimed to describe communication between neonatal health care professionals and parents in the presence of language barriers.

**Methods**: A field study using a hermeneutic lifeworld approach, participative observation, and interviews with parents and health care professionals.

**Results**: The main theme, *endeavouring to understand the meaning behind the words*, comprised three themes. *Wanting to speak for oneself* meant that parents wanted to speak for themselves or call on a friend or multilingual health care professionals, in contrast to the health care professionals wish to use an interpreter. *Being aware of cultural keys* meant that some wards had access to a “cultural broker” to assist health care professionals and parents with both language translation and understanding of the Swedish health care environment. *Understanding one another in the employees’ arena* reflected varying language skills among health care professionals. The health care professionals had the power to decide the level of access to communication, and decided both the intensity and the frequency of the conversations.

**Conclusions**: Health care professionals preferred to use an interpreter when communicating with parents, while parents wished to be independent and speak for themselves. If an interpreter was used, parents preferred this to be a friend or health care professionals; this option was less popular among health care professionals.

## Introduction

Family-centred care on the neonatal ward views parents and child as a single unit, and aims to support each family on the basis of its own specific needs. By listening to the parents’ life story, health care professionals can create trust between themselves and parents (Harrison, 2010).

Health care professionals in a Swedish study experienced cultural differences as challenging, and this made it hard for them to comprehend the wishes of families; for instance, that the spokesperson for the family was someone who visited the ward solely in order to make decisions regarding the care and treatment of the child (Patriksson, Nilsson, Berg, & Wigert, 2017). In another study, health care professionals realized that families from a different background were all unique, that people differed in their adherence to cultural norms, and that care therefore needed to be tailored to the individual (Hendson, Reis, & Nicholas, 2015). The social-cultural perspective included a lack of understanding of the importance of seeking early prenatal care and how to navigate the healthcare process. The parents experienced a feeling of shame and embarrassment due to the perception of their age or being unmarried during pregnancy. They also described perceived discrimination from prenatal care providers (Ayers et al., 2018).

A study of interaction between health care professionals and parents demonstrated that health care professionals were unprepared for or lacked training in handling social aspects around the family (Trajkovski, Schmied, Vickers, & Jackson, 2012). Other research has described situations where health care professionals believed that the parents spoke the local language, but language barriers became evident during conversation. Such barriers manifested themselves by, for example, consent being given in response to a question which had not been properly understood (Brooks et al., 2016).

A Swedish study showed parents of premature babies on a neonatal ward trying hard to identify something that was normal in their everyday life in a situation that was abnormal for the family, surrounded by strangers in an unfamiliar location. If there were siblings at home, the parents felt as if they were always in the wrong place. The parents stressed the importance of good communication, of believing that their worries about the child were taken seriously, and of being met with empathy. Opportunities for communication were reduced when the workload of health care professionals in the care unit was high. When parents on the neonatal ward were given opportunities to participate in the care of their child, their self-esteem was improved and they felt clearer about their role as parents. Their newly acquired knowledge provided a sense of security (Heinemann, Hellström-Westas, & Hedberg Nyqvist, 2013). Another study also confirmed that both parents and health care professionals felt that parents’ participation in the medical rounds was supportive (Abdel-Latif, Boswell, Broom, Smith, & Davis, 2015).

Language barriers have an impact on outcomes and health care delivery. Patients’ degree of satisfaction depends on how health care professionals respond to their communication needs (Squires, 2017). When health care professionals were unable to communicate with parents, they experienced frustration that the message they wanted to convey was not understood by the parents (Hendson et al., 2015; Patriksson et al., 2017). The use of the services of interpreters or bilingual health care professionals might contribute to greater patient satisfaction (Squires, 2017). A Swedish national mapping of the use of interpreters demonstrated a lack of guidelines for interpreted interviews, but health care professionals nevertheless rated their ability to carry out a conversation via an interpreter as good (Patriksson, Wigert, Berg, & Nilsson, 2019).

Studies have also shown that even if parents are able to speak and understand the local language, they may feel less stress if they can use their native language when difficult information has to be given and decisions have to be made (Azam Ali & Johnson, 2016). Health care professionals play a key role in supporting parents to feel calm even in stressful situations, and create a trustful relationship between them and the parents (Patriksson, Nilsson, & Wigert, 2019). However, these activities can become a challenge in the presence of language barriers. There is a lack of knowledge of how health care professionals and parents in neonatal care communicate when language barriers exist.

## Aim

The aim of this study was to describe communication between health care professionals and parents in the presence of language barriers.

## Method

This study was a field study using a hermeneutic lifeworld approach and involved participative observations and follow up interviews with parents as well as health care professionals; physicians, nurses and nurse assistants. The lifeworld approach offered the researchers a basis from which to analyze the world as experienced and communicated by people. The “lifeworld” is the world in which we lead our daily lives, and which is taken for granted. The research takes as its starting point descriptions of tangible, lived everyday experience (Dahlberg, Dahlberg, & Nyström, 2008). In the present study, this comprised the possibility of communication between parents and health care professionals and how this influenced parental participation in the care of their child.

Hermeneutic philosophy means having an open approach; this is the natural way in which we belong to the world, and the way of true understanding. Having an open mind enables us to see the otherness of something. The researcher is always connected with the past, but must be sensitive to the phenomenon being focused on and restrain their own pre-understanding (Gadamer, 2004).

## Context

The study was conducted at three selected neonatal units, one of which was a neonatal intensive care unit, in hospitals situated in the same Swedish region. These hospitals collaborate with each other, and neonatal patients might be transferred between units according to care needs and the availability of beds. In Sweden there are three levels of neonatal units, defined by level of care. Level I provides basic neonatal care, while level II represents specialist neonatal care. Level III is a subspecialty of neonatal intensive care, and is further subdivided into A–D, where D is full intensive care for extremely preterm babies (Stark, 2004). None of the hospitals in the study region provide neonatal care at level I.

All the neonatal units made it possible for parents to stay with their child. Whether this was in the same room as the child was dependent on the condition of the child and the facilities of the ward. One of the wards had a parent room adjacent to that of the child and operated a system of combined care, with the mothers being cared for by health care professionals from the maternity unit and the children being cared for by health care professionals from the neonatal unit. Some of the units had a cultural broker who clarified the differences between countries for health care professionals and parents. Neonatal wards differed as to whether the parents took care of the child on a 24-hour basis or only during the day. The neonatal intensive care unit at the university hospital treated seriously ill children, and hence had a high throughput of patients and a high workload. This unit had 23 beds and 130 health care professionals, while the other two units had 11 and 12 beds and 48 and 51 health care professionals, respectively. The duration of hospitalization varied from a few hours to several months, with mean durations of 8.2, 8.3, and 14.2 days respectively.

## Ethics

Permission to perform the study was requested from the heads of the units, and ethical approval and permission to undertake the study was obtained from the Regional Research Ethics Committee in Gothenburg, Sweden (ref: 665–17). The health care professionals in the three neonatal units were given spoken and written information about the study, while health care professionals and parents who were interviewed were personally briefed. All interviewees were assured that participation was voluntary, and that they were free to ask the observer to leave at any time. All information was treated in confidence, and the recorded and transcribed interviews were locked securely in a fireproof safe. The study complied with internationally-accepted ethical principles for medical research involving human subjects, designed to protect individuals and ensure respect for human dignity (WMA, 2013).

## Data collection

Data were collected over a three-month period in 2018 through participative observation and interviews with health care professionals and parents. Observations focused on the phenomenon of communication between health care professionals and parents in a neonatal unit, and were designed to identify both facilitating and obstructing factors. The data collector (first author, KP) was a specialist in paediatric nursing with 30 years of experience working in a neonatal unit. Her pre-understanding was influenced both by this experience and by the results of previous studies (Patriksson et al., 2017; Patriksson, Nilsson, et al., 2019; Patriksson, Wigert, et al., 2019). The data collector contacted each unit to verify if there were parents with language barriers. Field work was carried out over 62 h during, 52 observations and 20 interviews with health care professionals (10 interviews) and parents (10 interviews) on 32 different work shifts, including day, evening, and night shifts. The observations focused on communication between health care professionals and parents in the presence of language barriers.

The role of the data collector was to be a member of the health care professionals, but not to have any active share in the care of the child. The observer did not participate in communication between health care professionals and parents, except on rare occasions, such as if a direct question was asked. The observations were recorded as field notes, when possible during the actual observation or directly after it.

After some of the observations, health care professionals and parents were asked an open-ended question: “How did you feel in the care situation when you were not able to communicate in Swedish?”, with follow-up questions such as: “Can you give more detail about some specific situation?” The aim of the open-ended questions was to encourage parents and health care professionals to say more and to reflect on the possibility of communication.

In some cases, the follow-up consisted of subsequent interviews with parents (sometimes with a visiting friend of the parents acting as interpreter) and health care professionals in order to reach a deeper understanding of the phenomenon. Some of these interviews were recorded and transcribed verbatim, while others were reported in field notes, depending on the extent of the interview and where and when it took place. The selection of parents for interview was also influenced by their ability to communicate in the local language. Interviews with parents were carried out in Swedish, using very restricted language. If the parents did not understand, the question was reworded in simpler language with fewer words, with the aim of not altering the meaning. A total of twenty interviews were carried out, ten each with parents and with health care professionals.

## Data analysis

The analysis was carried out on texts from both the observations and the interviews, which were treated as a single text in accordance with the principles described by Dahlberg et al. (2008). The text was first read several times as a whole in order to gain familiarity with the data and a preliminary understanding of the phenomenon and its context. The analysis could be described as a movement from the whole to the parts and back again to the whole. During this movement, it was important to be sensitive to the whole, the data, and the phenomenon (Dahlberg et al., 2008). Preliminary subthemes and themes were constructed based on express meanings, so that the unknown could be understood in a new context. A preliminary interpretation was conducted to forge a new totality from the text, with great care being taken to identify latent meanings in the data. At the comparative level, a new interpretation was formulated to connect previous interpretations to each other. Semantic units were combined so as to make it possible to discern structures and patterns that were not previously readily apparent. There was an awareness that our preconceptions could affect our analysis (Dahlberg et al., 2008). Preconceptions come from prior experience, and it was important to maintain an awareness of this and restrain our preconceptions (Gadamer, 2004).

## Results

The results of the study can be summarized as the main theme: *endeavouring to understand the meaning behind the words*. This main theme was based on three themes: *wanting to speak for oneself, being aware of cultural keys*, and *understanding one another in the employees’ arena* (Table I).

**Table I.** Overview of subthemes, themes, and main theme.

Subthemes	Themes	Main theme
Being the parent of one’s child Being allowed to decide about an interpreter Withholding a right	Wanting to speak for oneself	Endeavouring to understand the meaning behind the words
Interpreting the codes Seeing what makes each family unique	Being aware of cultural keys	
Understanding each other in what is a foreign language to both Contributing to patient suffering resulting from nursing care Omitting information	Understanding one another in the employees’ arena	

In the following descriptions, quotations are used to illustrate the three themes, with the person quoted identified by one of the following labels: M = mother, F = father, N = registered nurse, and O = observer.

### Wanting to speak for oneself

The observations showed that it was important to have access to interpretation by a third person, in order to be able to communicate when there were language barriers between a member of health care professionals and a parent. The interpreter might be an authorized interpreter, a friend of the family who spoke the local language, or a health care professional who spoke the parents’ native language. Parents appreciated being allowed to decide who the interpreter should be. During observations, it was noted that parents were provided with an interpreter if they requested one. One father of a six-hour-old child made the comment that interpretation would be expensive for Swedish society in view of the large number of different languages, which led him to express a wish to manage without an interpreter. He said that he had learned about the high costs of interpretation via the television. Consequently, the father chose not to request an interpreter and therefore appreciated finding that a health care professional was fluent in his language.

“It’s better when it’s a doctor (who speaks the father’s language). I think I know. All this costs so much. I don’t pay anything, but it’s a cost for the municipality. It costs a lot.” (F)

In some cases, where multilingual members of health care professionals were used as interpreters, the parents would “light up” and immediately begin speaking in their native language and asking questions.

*“The physician spoke to the mother of a premature child in her native language, and communicated with her about the care and treatment of the child. This conversation was conducted without the use of an interpreter, and the mother smiled and answered the physician’s questions. The physician made clear to the mother that this conversation could only deal with purely medical matters and the care of the child, and the mother would not be able to raise any psychosocial issues concerning the family.”* (O).

After the medical round, the physician was interviewed and expressed the opinion that access to multilingual health care professionals was an asset. He understood the culture clash that occurred for parents who arrived in Sweden and then had a child needing care in a neonatal unit.

In all the neonatal units, it was usual for health care professionals not to involve interpreters in the first meeting with parents but postpone this to a later occasion. They saved all the information they wished to communicate to the parents for one occasion; a planned meeting where several different professionals might participate. This meant that parents received a great deal of information from several people on one occasion. The physician provided information about the medical care and treatment of the child, the nurse described the nursing care of the child, and finally the nurse assistant informed the parents about the routines of the ward.

One mother told a nurse that she wanted to communicate with the health care professionals in the local language in spite of her limited language skills, in order to learn the language. *“I also feel good about learning many, many words, for example nappy, mother’s milk, nipple shield, blanket. I didn’t know those words before. I want to talk. I want to understand everything too. Swedish is very hard.” (M)*

The nurse with whom the mother was talking expressed a need for materials such as pictures to use as support when communicating with parents, and suggested that such materials might be similar for all neonatal units.

One mother had been admitted urgently for treatment together with her newborn child, who was also in need of treatment. An authorized interpreter had been booked in advance in order to inform the parents about the condition of mother and child, and during the telephone-interpreted conversation, information was supplied by health care professionals from several different professions who were involved in the care and treatment of mother and child.

“The mother doesn’t ask any question, only listens, while the father, who is present, says that he doesn’t know whether he should be with his wife or his children. There are long discussions around how to solve this problem. Suddenly, in the middle of the conversation, the interpreter announces that the time is up, at which point the conversation has been running for about an hour. The nurse shouts out: ‘Wait, this is really important, we must extend the time.’ The father states via the interpreter that he wants to stay with his wife, but that there are other children at home and they need to come to the hospital so that the family can be together. The distance between the hospital and the family home is considerable, and the father has no possibility of collecting his children. Both the father and the health care professionals are trying to find a solution to the problem in order to make the situation as good as possible for the family.” (O)

After this conversation, the nurse pointed out in an interview how difficult it was to know what was possible in terms of help and support for the family when there were no guidelines about, for instance, taxi trips for a family member.

Parents were sometimes invited to participate in the discussions that took place during the medical rounds, in order to receive information about the care and treatment of their child and to take part in the care of their child. The information the parents received was often given in Swedish and there was no-one present who could interpret for them. On one occasion, a family member was present as interpreter for the parents to make it possible for them to ask questions about the care and treatment of their child.

“The doctor comes into the patient’s room and tells the parents and the mother’s brother ‘I’m going to take your child on my rounds and you need to follow along with the nurse and me.’ The parents and the brother follow the doctor into the office area and the doctor immediately asks them ‘How do you think your child is feeling?’ The brother interprets and the parents receive the information they have been expecting. The round is over quickly and parents and the brother go back to the room and talk to the nurse who has stayed in the room, and the brother says how nice it was that he could be included and serve as interpreter for his sister.” (O).

Some of the mothers had a woman friend with them on the neonatal ward, principally when it was impossible for the father to stay. Usually the friend would speak the local language and hence could act as support for the mother. One mother, who was tired and spent much of her time resting in bed, had a visit from a woman friend and ended up sitting in an armchair with a smile on her face, eating a meal together with her friend, and having a lively conversation with her.

On some occasions when health care professionals communicated with parents without an interpreter, they made use of pictorial support to supplement the spoken conversation.

“One mother came with her newborn child from another hospital, for care in the regional hospital, and is anxious because her child has had a major operation. She is afraid of approaching the child and is standing aside, looking at it. The nurse assistant who is looking after the child uses pictorial support, and at the same time keeps smiling at the mother. Now the mother, with the support of the nurse assistant, tentatively takes part in the care of her child and the nurse assistant encourages the mother to kiss her child. When the mother leans over the child she is smiling, while tears run down her cheeks.” (O)

At a subsequent interview with the nurse assistant, she expressed the opinion that it was always possible to communicate in one way or another, provided there was time available to spend with the parents.

### Being aware of cultural keys

The parents on these wards came from different countries and cultures. The observations showed that there were cultural brokers whose task it was to act as a bridge between health care professionals and parents in everyday conversation, and to improve their mutual understanding.

One nurse stated in an interview that attitudes to breastfeeding, colostrum, and the period immediately after the birth varied from one culture to the next, but that there was a cultural broker who clarified the differences between countries for both health care professionals and parents. This could be seen as positive by the health care professionals, and helped them support the family in the daily care of the child.

“She’s afraid of pronouncing words because she might get them wrong, for instance ‘ropa’ (shout) when she wants to say ‘rapa’ (burp). We have a guide here who helps us explain how different cultures view different things, such as the idea that the colostrum might be regarded as dirty.” (N)

Sometimes there were health care professionals on the floor who were fluent in several languages including the parents’ native language, which the parents appreciated. Being able to speak and ask questions in their native language meant that the parents had a lot to say. They did not want to end their conversations with the health care professionals.

“During a medical round it’s reported that one set of parents are anxious and don’t want to participate in the care of their child. Several different solutions are proposed for the health care professionals to be able to get the family to be active in the care of their child. One nurse assistant who has been working during the day, and who speaks the family’s native language, goes to talk with the parents in their common language. When she enters their room and the parents understand that the nurse who helped them look after the child speaks their language, they are pleased and express their fear in relation to their new parenthood. The nurse assistant explains clearly what she is about to do with the child, but when this is carried out the father gets very agitated. When the mother comes to the changing table and wants to see what is happening to her child, the father asks her to leave. The nurse assistant starts removing the peripheral venous catheter in the child’s arm, and the boy starts crying and is hungry. The father sighs again and sends the mother out of the room, saying ‘She shouldn’t have to see this.’ Then he activates the alarm and says ‘We must have more health care professionals.’” (O)

In a subsequent interview with the mother, she recounted (through the father) that her sister would come and take care of the child for the initial period at home and that she was concentrating on resting now, while she was in the hospital. Health care professionals had been concerned about the mother is resting during large parts of the day, but nobody had spoken to the mother about this.

### Understanding one another in the employees’ arena

The health care professionals had the power to decide the level of access to communication, and they decided both the intensity and the frequency of the conversations. Most of the parents who had knowledge of English felt that they could speak for themselves in English. They wished to speak English with the health care professionals, whose knowledge of the English language was variable. In subsequent conversations with the health care professionals the professionals expressed a fear of speaking English with the parents and using the wrong words at the wrong time. The health care professionals decided that access to English conversations (i.e., the health care professionals English skills) could influence the possibility for the parents to communicate on the wards.

“One parent on a ward is anxious about the child and tries to contact the health care professionals, addressing them in English. The health care professionals present on the ward make pushing-away gestures, shake their heads and say ‘No, no.’” (O)

The nurses also decided when and whether there would be a conversation with the parents using an interpreter. This meant that it could be several days before the parents received information about their child’s status, or that the parents misunderstood the situation. The parents could get the impression that their child’s life was in danger when it was not, or that the parents themselves had caused the child’s illness or condition. This created a situation of entirely unnecessary suffering on the part of the parents, caused by the health care professionals.

“One set of parents are told at the medical round that their child has a bacterium, but that this will not cause any problems for the child. The parents become very anxious, and see signs of illness in the child when these are simply natural for a prematurely born child. During the next shift, the parents are talking with the health care professionals about their belief that their child is seriously ill. They want to give the child a bath, to keep his skin clean and thus avoid bacteria entering via the skin. On the following day, the parents are informed about the child’s bacterium through an interpreter, and they then enter the child’s room. They can’t see that the child looks good and has gained weight, and are anxious. The father tells the health care professionals that they want to give their child a bath to keep him as clean as possible so that no bacteria will enter through some sore, which the physician had said might happen.” (O)

Another source of suffering resulting from nursing care was the poor telecommunication equipment on the wards. Sometimes interpreted conversations were held using a telephone not designed for telephone interpretation, and the sound quality was so poor that it was difficult to hear what was being said. It was difficult to carry on multi-party conversations by telephone, and misunderstandings often arose.

“A conversation between parents and health care professionals is taking place via telephone interpretation. The health care professionals are speaking clearly and in short sentences. Suddenly the interpreter interrupts the conversation and makes clear that he does not quite understand what it is that is to be put across. The telephone in use is an ordinary one, and the sound quality is variable. The conversation is taking place with all those present standing round the telephone so that they will all be able to hear what is said in the course of the conversation. The physician is informing the mother of why the child is being cared for in the unit. He does so in easy Swedish and draws parallels with blood and sour milk to help the mother understand high levels of erythrocytes.” (O)

The health care professional’s documentation of the parents’ communication needs could have an impact on the availability of interpreters during these conversations. The health care professionals stated that there was usually no documentation of whether the parents needed interpretation, and that this would have to become clear from contact with the parents. On one ward, there was a set of parents who usually were not present when elements of care such as turning over or changing nappies were about to take place. The nurse stated that the health care professionals were unable to communicate with these parents, which meant that it was impossible for the health care professionals to plan the day based on the child’s care needs.

“It’s a pity that they miss out on so much of the care.” (N)

## Main interpretation and discussion

From the three themes analyzed above, a main interpretation emerged of how to clarify the conditions for communication between health care professionals and parents whose child is cared for in a neonatal unit. There was a discrepancy between parents’ and health care professional’s views of an ideal communication situation. While the health care professionals asked for skilled interpreters who understood medical terminology, the parents thought that trust was the most important aspect, and wanted to speak for themselves. If an interpreter was to be present, the parents preferred this to be a friend or another member of the health care profesionals.

The health care professionals felt that parents who were unable to communicate in the local language were in a difficult situation. They wished that they had further means of facilitating communication, other than regular conversations through an interpreter. An interpreter was always contacted if parents asked to use one during conversations, but it was up to the parents themselves to determine whether they needed an interpreter.

The health care professionals were not aware of any financial restrictions on when and how they were allowed to make use of interpreters, and stated that parents had regular conversations through an interpreter. However, the observations showed that conversations through an interpreter were rare and often time-limited. Misunderstandings between parents and health care professionals easily arose in the course of the daily care of the child, which then led the parents to become anxious. Some parents who spoke English wanted to communicate in English, which some of the health care professionals found difficult due to their limited knowledge of the language. One health care professionals stated that she felt uncomfortable speaking English because she rarely used English words in daily life.

The neonatal units made it possible for parents to stay with their child. Sometimes parents stated that they did not dare to participate in the care of their child, or the mother handed this over to the father. Language barriers put parents at a disadvantage due to not knowing that parental participation in the care of their child was encouraged in these units. In a previous study (Hendson et al., 2015), health care professionals and providers seeking to understand other people’s perspectives described strong feelings of empathy for the new immigrant family, and emphasized the importance of stepping back, being humble, and listening to the views of the families. Whether because of language barriers or cultural differences, these professionals believed that more time was required to form relationships with new immigrant families and to teach them about the care of their infant (Hendson et al., 2015). The parents in the present study stated that if an interpreter was needed, they preferred multilingual health care professionals. They expressed concern about the cost of interpreters for the health care system, but appreciated meeting a person who spoke their language. Bilingual health care professionals understand the medical terminology of the language they speak and speak the language well enough to communicate with the families (Betancourt, Renfrew, Green, Lopez, & Wasserman, 2012).

Being multilingual increases the work load for health care professionals, since they have to carry out their own work at the same time as acting as interpreter for a colleague (Azam Ali & Johnson, 2016). Studies also show that health care professionals do not have training in interpretation, which might lead to poor quality translation (Hadziabdic & Hjelm, 2014). On one occasion observed in the present study, there was a great deal of information to give to the parents, but since the interpreter appointment was time-limited there was no opportunity for the parents to ask questions. In the end, it was possible to extend the time for the interpreted conversation, but this was due to the interpreter’s generosity and concern for the parents.

One neonatal unit used a cultural broker who was tasked with acting as a bridge between health care professionals and parents. The health care professionals found it very helpful during conversations to be able to get an explanation of what was different in different cultures, as well as having a cultural broker who was able to translate in care situations. A cultural broker is an active participant who helps health care professionals understand attitudes within the parents’ culture and helps parents understand Swedish culture, thus bridging the gap between them (Akkerman & Bakker, 2011). This interpretation service model was developed from the understanding that communication has serious weaknesses if attention is not paid to the role of culture in the substance and style of the communication (Lee, 2000).

On some occasions, parents were invited to participate in the medical round and discussion of the child, were asked about their child’s condition, and were able to express their opinion about their child’s condition. When there were language barriers, the parents were given the opportunity to have a family member present as interpreter. It was difficult to pre-book an interpreter, since the timing of the medical discussion was dependent on the care burden of the unit, and it was difficult to set an exact time.

Physicians can support parental participation in decision making during medical rounds by asking the parents how they view their child’s condition. The parents are able to express how their child is doing, and the neonatologist can express agreement and understanding both verbally and through non-verbal communication such as nodding and smiling (Axelin, Outinen, Lainema, Lehtonen, & Franck, 2018). One study showed that parents who were allowed to participate in the medical round, and were given the opportunity to take decisions concerning their child, felt that they were closer to their child (Treherne, Feeley, Charbonneau, & Axelin, 2017).

One mother in the present study did not dare touch her son, who was very ill, and stood there looking at him with tears in her eyes. Because the paediatric nurse took her time and tried to reach the mother by making her participate, the mother smiled again and dared to touch her son. All the time, the nurse assistant spoke calmly with the mother and used pictures as support when she could see that there was something that the mother did not understand. The use of pictorial support is a complement to body language, but it can also be used for clarification, for instance during telephone interpretation (KomHIT, 2016).

## Methodological reflections

The research process used in this field study has both limitations and strengths. One limitation might be that the data collector (KP) was an expert on the subject matter of the study, and there was a risk of falling out of the role of observer and acting like a nurse. To understand a phenomenon, it is important to be able to be both distant and close, sometimes at the same time (Dahlberg et al., 2008). At the same time, it is a strength of this study that the first and last authors (KP & HW) were familiar with the environment of a neonatal unit. It was important to have an awareness of pre-understanding and openness, closeness, and distance to the studied phenomenon, namely communication in a neonatal unit. It was not possible to be entirely objective as a researcher, but it was necessary to restrain the pre-understanding.

## Conclusions

The health care professionals in this study preferred to communicate with parents with the aid of an interpreter, but the parents wished to be independent of interpreters and speak for themselves. If an interpreter was to be present, the parents preferred this to be a friend or another member of the health care professionals but this option was less popular among the health care professionals. Several instances of misunderstanding arose due to the health care professionals not using an interpreter when communicating with the parents, which resulted in unnecessary suffering on the part of the parents, caused by the nursing care. When parents felt that the use of an interpreter made them into a financial burden, they were prevented from getting information about their child. The joy experienced by parents when multilingual health care professionals were used as interpreters illustrates that it is not just the translation of words that is desired, but also a feeling of cultural community. It is possible that improved knowledge about the parental experience of having a child in the care of a neonatal unit might help give parents early knowledge about the care and treatment of their child, and provide health care professionals with more knowledge about how to interact with parents in the presence of language barriers.
